# A rare case of lepromatous leprosy in Germany

**DOI:** 10.1111/ddg.15801

**Published:** 2025-06-17

**Authors:** Roman Saternus, Gisela Bretzel, Tamaz Tsulaia, Kristin Burckhardt, Krista Yordanova, Sophie Elisabeth Müller, Steffen Jahn, Thomas Vogt, Sophie Schneitler

**Affiliations:** ^1^ Klinik für Dermatologie Venerologie und Allergologie am Universitätsklinikum des Saarlandes, Homburg; ^2^ Institut für Infektions‐ und Tropenmedizin am Klinikum der Universität München; ^3^ Institut für Medizinische Mikrobiologie und Hygiene am Universitätsklinikum des Saarlandes, Homburg; ^4^ Pathologie an der Dresdner Heide, Radeberg

Dear Editors,

A 56‐year‐old male patient presented to our university outpatient clinic for the first time. He reported having progressively growing, distressing lesions on his face and distal forearms for several months. He described reduced sensitivity within the affected areas. The patient, originally from Ghana, has been living in Germany for an extended but unspecified period of time, working as a hotel cleaner. He had previously been treated by dermatologists and ENT specialists under the working diagnosis of rhinophyma, but without clinical improvement. Beside a monoclonal IgG gammopathy of unclear significance, there was no record of previous illnesses or ongoing long‐term medication.

Symmetrical, hypesthetic, confluent flesh‐colored papules and nodules without epidermal changes were observed centrofacially, involving the forehead, nose, cheek, and lip. There was also rarefaction of the lateral eyebrows (Figure 1). On the distal forearms, individual, ulnar‐sided, subcutaneous, indolent nodules were present.

**FIGURE 1:**
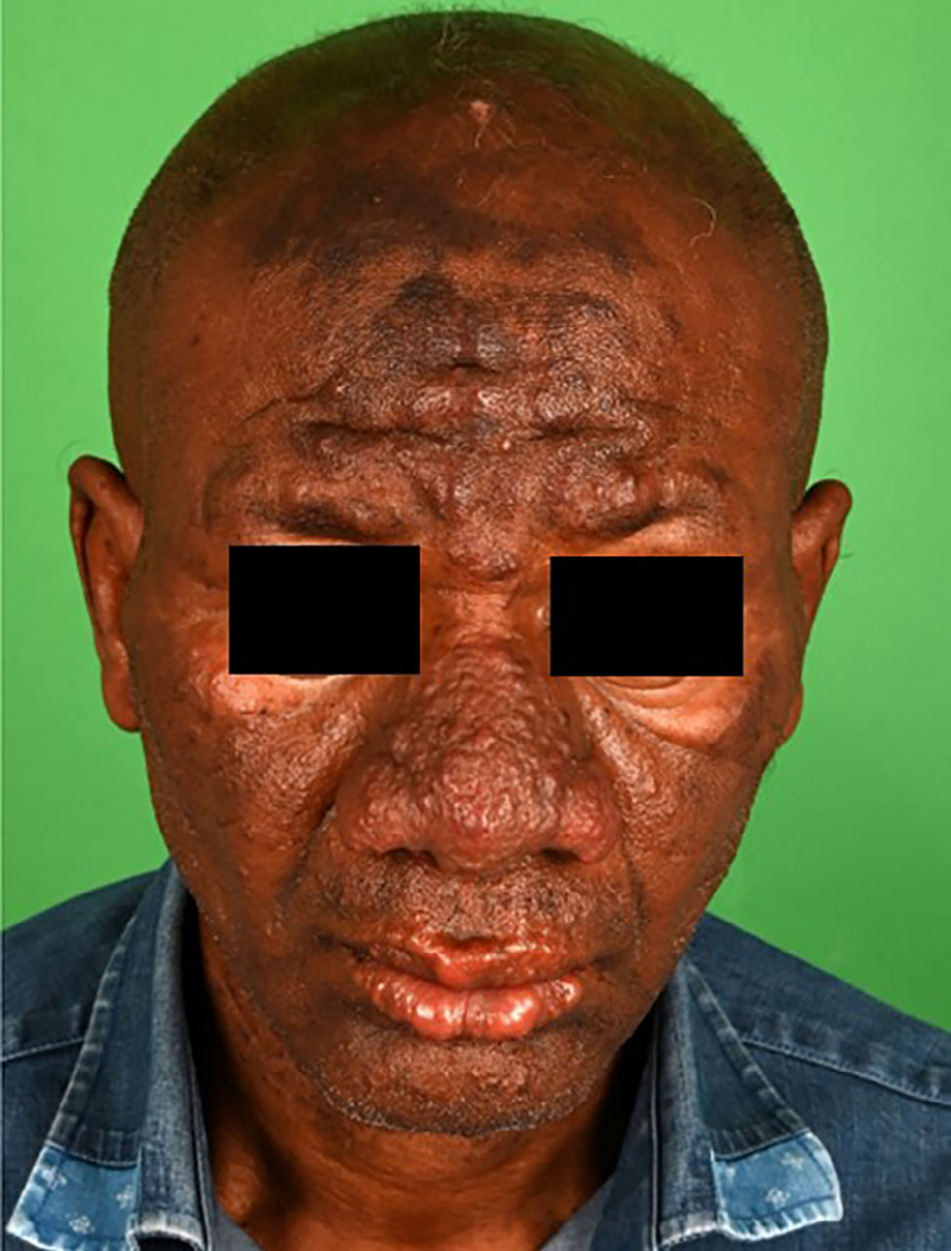
Skin lesions at first presentation.

Multibacillary lepromatous leprosy was suspected, associated with the typical leonine facies. Further diagnostic workup was immediately initiated, and the suspected case was reported to the responsible health authorities.

Using RLEP‐Real‐Time‐qPCR, *Mycobacterium leprae* could be detected in swab material from the nasal mucosa and in two skin biopsies from the forehead, with a high bacterial load (nasal mucosa: approx. 35,000 bacteria in 50 µL DNA extract; Skin biopsies: approx. 668,000 and 750,000 bacteria in 50 µL DNA extract). A high PGL‐1 titer was found in serology. The additional conventional histopathological examination of another skin biopsy from the forehead revealed a pandermal, dense histiocytic infiltrate with foamy macrophages (Virchow cells) without epidermal changes. There were no giant cells.

Using Fite‐Faraco staining, large numbers of intracellular and in some cases interstitial rod bacteria were detected (Figure 2). This unambiguously confirmed the suspected diagnosis of leprosy. Secondary findings indicated a lapsed hepatitis B, but there was no serological evidence for hepatitis C or HIV infection.

**FIGURE 2:**
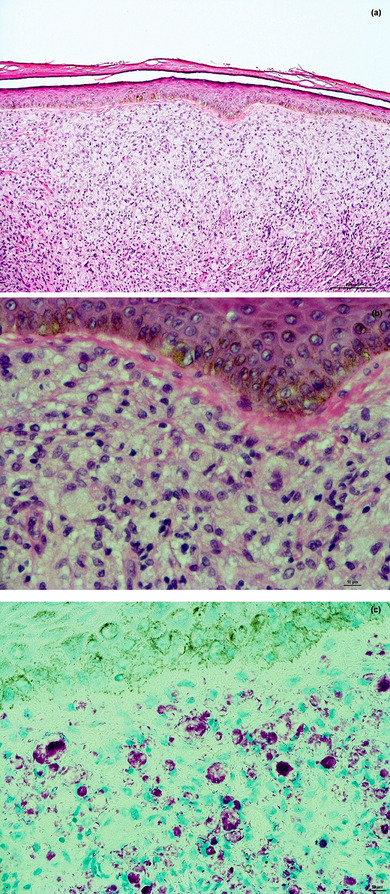
(a) Histopathology of a biopsy from the forehead (hematoxylin‐eosin stain [HE], original magnification x 100). A pandermal histiocytic infiltrate is present without multinucleated giant cells. The macrophages show large, foamy cytoplasm (so‐called Virchow cells). No epidermal involvement. (b) HE staining, x 400. (c) Fite‐Faraco staining (original magnification x 400). Numerous pink‐violet, intracellular and interstitial rod‐shaped bacteria are visible. The epidermis is not involved.

The one‐year triple antibiotic therapy recommended by the World Health Organization, consisting of clofazimine, rifampicin, and dapsone (Table 1), was initiated just a few days after the initial presentation, using clofazimine obtained from abroad.^1^ Complicating matters further was the fact that the patient's residence status and identity had since become unclear, meaning that in addition to infection hygiene issues, intensive discussions with the authorities also had to focus on ensuring treatment adherence and, last but not least, the coverage of costs in the absence of health insurance. So far, the therapy has been tolerated without side effects or leprosy reactions. The effects of the monoclonal IgG gammopathy of unclear significance mentioned in the case history remain to be seen. To date, only isolated case reports on this topic have been described in the literature.^2^, ^3^

**TABLE 1:** Treatment as recommended by WHO.^1^

Antibiotic	Monthly single dose	Daily dose
Rifampicin	600 mg p.o.	–
Clofazimine	300 mg p.o.	50 mg p.o.
Dapsone	–	100 mg p.o.

Leprosy is one of the oldest known diseases and has accompanied mankind for thousands of years. Studies have shown that the DNA of *Mycobacterium leprae* has remained largely conserved for centuries.^4^ Genetic factors, for example in the HLA expression pattern, appear to play a decisive role in whether an exposed person contracts leprosy.^5^ These factors, together with good hygiene standards and a well‐developed healthcare system, have rendered the disease rare in Europe – contrasting with the very high case numbers recorded in the Middle Ages. In Germany, a maximum of not more than five cases per year were registered between 2001 and 2015.^6^ Most cases involve migration‐associated infections. However, owing to the occasionally limited hygienic conditions and low socio‐economic living standards in this context, direct transmission modes cannot be completely ruled out.^7^ Around 95% of leprosy cases now occur in the Global South.^8^ According to the World Health Organization, there was a downward trend in newly diagnosed leprosy cases worldwide between 2006 and 2016.^1^

As the bacteria are present in the nasal mucosa, transmission is probably aerogenic and via contact infection.^9^ The incubation period is unusually long and can last 3 to 7 years, in individual cases even several decades, giving leprosy the longest known incubation period of a human infectious disease.^6^ *Mycobacterium leprae* initially multiplies in macrophages of the nasal mucosa after which, through the bloodstream, it finally reaches the Schwann cells of the peripheral nerve myelin sheaths.^6^ Macrophages attempt to eliminate the pathogen. The infected macrophages are referred to as foam cells due to the formation of lipid‐filled phagosomes, or as Virchow cells after Rudolf Virchow, who first described them.^6^ Depending on the immune status of the infected person, the disease manifests itself in different stages, which differ significantly both clinically and by histopathologically. These include the two polarized forms of leprosy: tuberculoid (paucibacillary) leprosy, which is based on a Th1‐dominated cellular immune response, and lepromatous (multibacillary) leprosy, in which a Th2 immune response predominates.^6^, ^10^ Transitions exist between these maximally polarized forms of leprosy, referred to as borderline leprosy (borderline tuberculoid, borderline borderline and borderline lepromatous leprosy).^6^

In multibacillary leprosy, the suspected clinical diagnosis can usually be quickly confirmed by PCR testing of tissue samples and nasal swabs.^11^, ^12^ In paucibacillary leprosy, the sensitivity of PCR testing is lower, so confirming the diagnosis can often be challenging.^13^ Through histopathological examination, tuberculoid leprosy usually presents with granulomas of epithelioid cells and giant cells with an accompanying lymphocytic infiltrate. In borderline leprosy, the granulomas become increasingly disorganized with increasing lepromatous polarization, with a decrease in epithelioid cells, giant cells, and lymphocytes, while the number of histiocytes increases. The full picture of lepromatous polarized leprosy ultimately emerges, as in our case.^14^

This case impressively shows the typical clinical and histological picture of multibacillary leprosy, which is extremely rare in Europe. In light of migratory movements to Europe, it also illustrates how essential the knowledge of rare infectious diseases can be for accurate and rapid diagnosis and thus for treatment initiation.

## CONFLICT OF INTEREST STATEMENT

R.S. recieved speaker’s fee and/or travel support from MedKom Akademie, KYOWA Kirin, Lilly, Eucerin, Unna Akademie, RG Gesellschaft für Information und Organisation, Sun Pharmaceutical Industries, Boehringer‐Ingelheim, Galderma, Pfizer and Novartis.
